# 

**Published:** 1904-12

**Authors:** 


Twentieth Year.

Vol. XX. DECEMBER, 1904. No. 6.

[illegible]
DEC. 12. 1904

### TEXAS Medical Journal.

Subscription, $1.00 a Year, in Advance.

Single Copies, 10 Cents.

PUBLISHED AT AUSTIN, TEXAS, BY

F. E. DANIEL, M. D.,

Proprietor.

PRINTED BY THE VON BOECKMANN-JONES CO.

Entered at the Postoffice at Austin, Texas, as Second-class Mail Matter.

